# The SMYD3-dependent H3K4me3 status of IGF2 intensifies local Th2 differentiation in CRSwNP via positive feedback

**DOI:** 10.1186/s12964-023-01375-y

**Published:** 2023-11-30

**Authors:** Lei Yu, Yi Wei, Tong Lu, Zhengqi Li, Shimin Lai, Yan Yan, Changhui Chen, Weiping Wen

**Affiliations:** 1 Department of Otolaryngology, The Sixth Affiliated Hospital, Sun Yat-sen University, Guangzhou, Guangdong P.R. China; 2 Department of Otolaryngology, The First Affiliated Hospital of Sun Yat-Sen University, Guangzhou, Guangdong P.R. China; 3 Otorhinolaryngology Institute of Sun Yat-Sen University, Guangzhou, Guangdong P.R. China; 4 Guangzhou Key Laboratory of Otorhinolaryngology, Guangzhou, Guangdong P.R. China

**Keywords:** H3K4me3, SMYD3, IGF2, Th2 CRSwNP

## Abstract

Chronic rhinosinusitis with nasal polyps (CRSwNP) is a heterogeneous and common upper airway disease divided into various inflammatory endotypes. Recent epidemiological findings showed a T helper 2 (Th2)-skewed dominance in CRSwNP patients. Histone modification alterations can regulate transcriptional and translational expression, resulting in abnormal pathogenic changes and the occurrence of diseases. Trimethylation of histone H3 lysine 4 (H3K4me3) is considered an activator of gene expression through modulation of accessibility for transcription, which is closely related to CRSwNP. H3K4me3 levels in the human nasal epithelium may change under Th2-biased inflammatory conditions, resulting in exaggerated local nasal Th2 responses via the regulation of naïve CD4^+^ T-cell differentiation. Here, we revealed that the level of SET and MYND domain-containing protein 3 (SMYD3)-mediated H3K4me3 was increased in NPs from Th2 CRSwNP patients compared with those from healthy controls. We demonstrated that SMYD3-mediated H3K4me3 is increased in human nasal epithelial cells under Th2-biased inflammatory conditions via S-adenosyl-L-methionine (SAM) production and further found that the H3K4me3^high^ status of insulin-like growth factor 2 (IGF2) produced in primary human nasal epithelial cells could promote naïve CD4^+^ T-cell differentiation into Th2 cells. Moreover, we found that SAM production was dependent on the c-Myc/methionine adenosyltransferase 2A (MAT2A) axis in the nasal epithelium. Understanding histone modifications in the nasal epithelium has immense potential utility in the development of novel classes of therapeutics targeting Th2 polarization in Th2 CRSwNP.

Video Abstract

**Supplementary Information:**

The online version contains supplementary material available at 10.1186/s12964-023-01375-y.

## Introduction

Chronic rhinosinusitis with nasal polyps (CRSwNP) is a complex respiratory disease characterized by heterogeneous airway inflammation. Local inflammation and immune responses in the nasal mucosa are responsible for the pathophysiological features of the endotype classifications of CRSwNP, which include type 1, type 2, and type 3 [1–3]. Given that the T helper 2 (Th2)-skewed inflammatory endotype of CRSwNP (Th2 CRSwNP) is closely associated with refractoriness and comorbidities and is highly prevalent [4, 5], it has garnered more attention. From a pathophysiological perspective, Th2 CRSwNP is notably characterized by local Th2 inflammation and immune dominance in the nasal mucosa, activation and recruitment of Th2 cells and of high local production of Th2 cytokines, including Interleukin (IL)-4, IL-5 and IL-13 [6–8]. The differentiation of naïve CD4^+^ T cells into various lineages of Th cells is crucial in many immune diseases [9], and Th2 cell differentiation from naïve CD4 + T cells contributes to the onset of Th2 CRSwNP. However, the specific interaction mechanisms and regulatory factors involved in this process are incompletely understood and require further elucidation. Given the crucial role of the nasal epithelium as the first line of defence against hazards and pathogens, any abnormalities in the nasal epithelium can result in pathogenic changes within both the epithelium itself and the surrounding microenvironment**.** The nasal epithelium has been widely recognized as a key factor in the pathogenesis of CRSwNP since the late 1990s. Histone modifications are major epigenetic modifications with crucial regulatory effects on gene transcription [10] and participate in many human diseases. Based on advanced techniques and extensive data on histone modifications [11–14], pathologic changes in airway diseases, including inflammatory factor production, immune responses and remodelling processes, have been proven to be closely correlated with changes in histone modifications in the airway epithelium. Among various histone modifications, trimethylation of histone H3 lysine 4 (H3K4me3) has been extensively studied and shown to be closely associated with transcriptional activation, and it is particularly located near transcription start sites (TSSs) [15, 16]. SET and MYND domain-containing protein 3 (SMYD3) has been identified as a vital methyltransferase responsible for H3K4me3 [17–19]. SMYD3 plays a role in promoting gene expression and contains two critical binding regions, namely, S-adenosyl-L-methionine (SAM)- and substrate-binding pockets [20]. Additionally, the transfer of methyl groups from SAM to histones via specific histone methyltransferases (HMTs) has been verified to be a vital process for the activation of gene expression through H3K4me3. IL-4-driven SMYD3-mediated H3K4me3 depends on SAM production, as SMYD3 is a SAM-dependent enzyme. Notably, plasticity of the human nasal epithelium in a Th2-biased inflammatory environment can lead to changes in histone modification levels. In this process, the activity of upstream and downstream HMTs as key mediators connecting Th2 stimulation and H3K4me3 attracted our attention. Insulin-like growth factor 2 (IGF2), considered a regulator of Th differentiation and transcription, is controlled by epigenetic marks on differentially methylated regions (DMRs) [21, 22], suggesting that the H3K4me3 status of IGF2 might be a key factor affecting the Th-biased microenvironment.

We hypothesize that H3K4me3 modifications in the nasal epithelium, which regulate transcriptional activity during Th2 CRSwNP development, are closely linked with the local immune response. Subsequently, we validated our hypothesis through in vitro experiments, showing the patterns in nasal epithelial cells in a Th2 cytokine-predominant microenvironment were similar to those in tissues. Furthermore, we elucidated the pathway through which Th2 cytokines interfere with the SMYD3-mediated H3K4me3^high^ status, thereby exerting a promoting effect on Th2 differentiation via IGF2.

## Results

### Aberrant global H3K4me3 levels and specific methyltransferases-SMYD3 among Th2-CRSwNP, non-Th2 CRSwNP and control groups

**Fig. 1:**
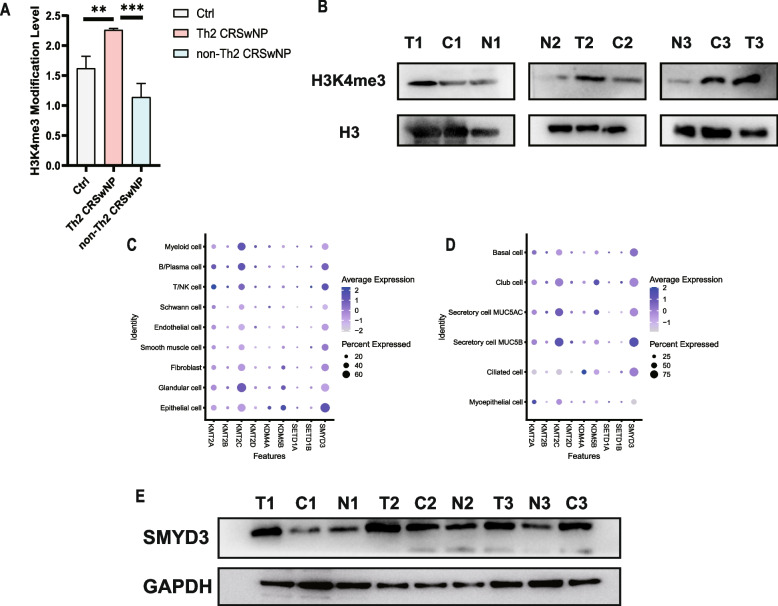
Elevated levels of SMYD3-mediated H3K4me3 in the Th2-CRSwNP group but decreased levels in the non-Th2 CRSwNP group compared with the control group. **A**-**B** Histone H3 modification assay (**A**) and immunoblot analysis (**B**) of H3K4me3 in the three groups (control, Th2 CRSwNP and non-Th2 CRSwNP). H3 was used as a loading control for Western blotting. (*n* = 3/group). Statistical significance, one-way ANOVA;***p < 0.001;**p < 0.01. T: Th2 nasal polyps; C: control; N: non-Th2 nasal polyps. **C** and **D** Single-cell analysis of the distribution of specific H3K4me3-related enzymes (KMT2A, KMT2B, KMT2C, KMT2D, KDM4A, KDM5B, SETD1A, SETD1B and SMYD3) in various types of cells and in subgroups of epithelial cells. **E** Immunoblot analysis of SMYD3 expression in the three groups described in (**B**). GAPDH was used as a loading control for Western blotting..T: Th2 nasal polyps; C: control; N: non-Th2 nasal polyps (*n* = 3/group)

To explore the role of H3K4me3 modification remodelling in Th2 CRSwNP development, we first assessed the levels of H3K4me3 in NP tissues from Th2-CRSwNP and non-Th2 CRSwNP patients (*n* = 3/group) and inferior turbinate (IT) tissues from control individuals (*n* = 3) by a H3 modification assay. The global H3K4me3 level was significantly increased in NP tissues in the Th2 CRSwNP group but decreased in the non-Th2 CRSwNP group compared with the control group (Fig. 1a). Additionally, Western blot analysis of H3K4me3 revealed a modification pattern comparable to that shown above (Fig. 1b). To determine the HMT responsible for H3K4me3 in the human nasal epithelium, we performed single-cell sequencing to profile H3K4me3-related enzymes. SMYD3 displayed a higher expression level and percentage than any of the other eight enzymes evaluated (Fig. 1c). Furthermore, SMYD3 was predominantly expressed in secretory and club cells (Fig. 1d). Subsequently, SMYD3 protein expression in all participants was measured and found to show a trend similar to that of the changes in H3K4me3 (Fig. 1e). Collectively, these data indicate that the SMYD3-mediated H3K4me3high status may play an essential role in Th2 CRSwNP development.

### Altered levels of H3K4me3 and SMYD3 in primary human nasal epithelial cells (HNECs) in the Th2-dominant microenvironment

**Fig. 2:**
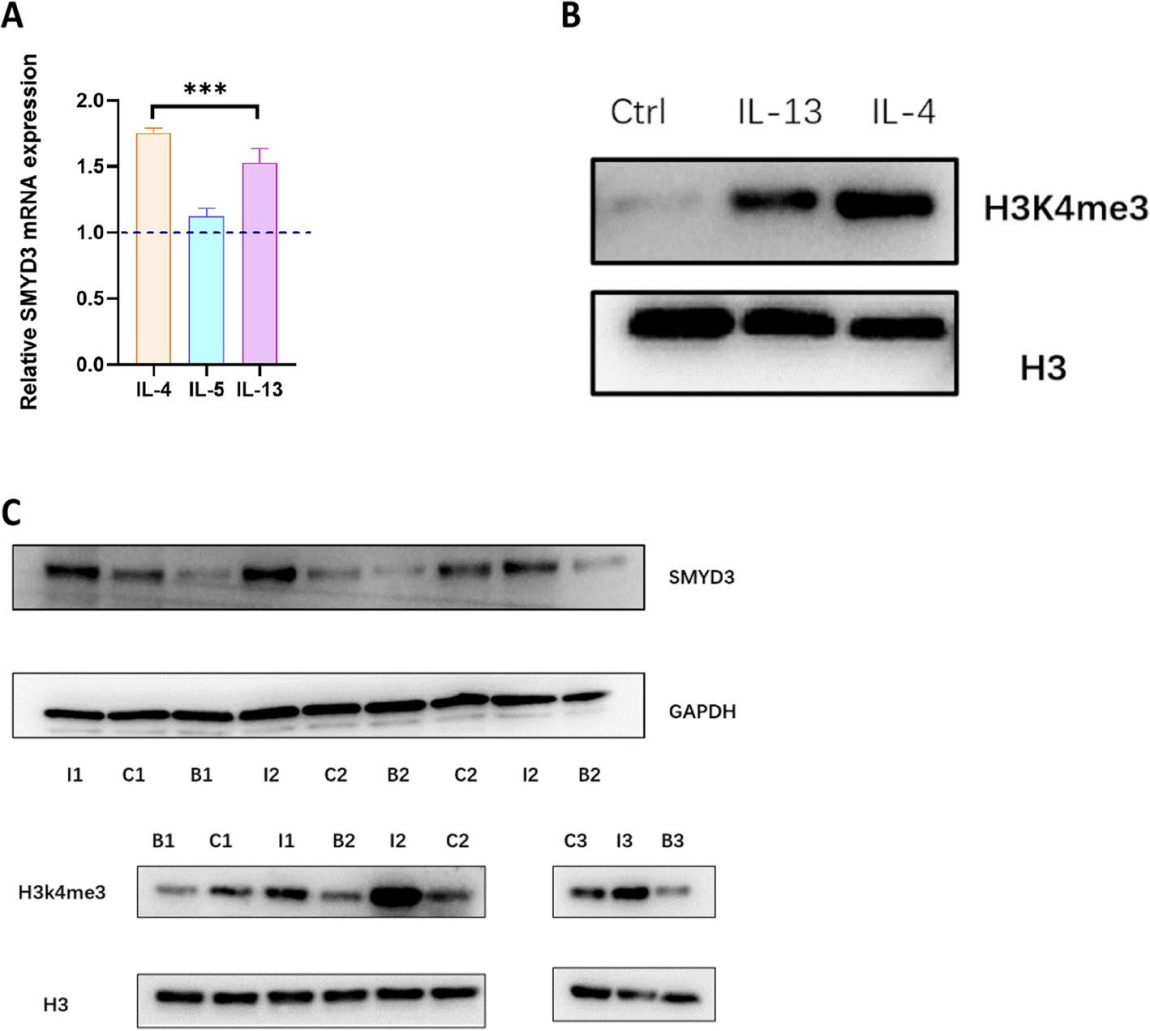
IL-4 stimulation induces robust SMYD3-mediated H3K4me3 modification in primary human nasal epithelial cells, and treatment with an SMYD3 inhibitor reduces this effect. **A** Quantitative RT‒PCR analysis results showing increased transcript levels of SMYD3 in human nasal epithelial cells from controls (untreated and treated with various cytokines (IL-4, IL-5 and IL-13)) (*n* = 3). Statistical significance, one-way ANOVA;*** p < 0.001. **B** Western blot analysis of H3K4me3 levels in human nasal epithelial cells treated with IL-13 or IL-4. **C** Immunoblot analysis of SMYD3 and H3K4me3 levels in primary human nasal epithelial cells stimulated with IL-4 and IL-4 + BCI-121 (50 μM) cultured in the ALI system (*n* = 3). I: IL-4 stimulation; **C**: control; **B**: IL-4 stimulation + BCI-121 treatment

We used typical Th2 cytokines (including IL-4, IL-5 and IL-13) to stimulate primary human nasal epithelial cells and found that compared with IL-5 and IL-13, IL-4 showed a significant and strong positive correlation with H3K4me3 modification and SMYD3 expression (Fig. 2a, b). Therefore, nasal epithelial cells were pretreated with IL-4 (10 ng/mL) to mimic the Th2 milieu in vitro. The protein levels of H3K4me3 and SMYD3 were then measured by immunoblotting, and IL-4 was found to lead to high levels of H3K4me3 and SMYD3 expression. To validate the causal correlation between SMYD3 and H3K4me3, we employed BCI-121 as a small molecule inhibitor of SMYD3. BCI-121 specifically targets the substrate pocket of SMYD3, thereby inhibiting its activity [19, 23]. Consistent with the above results, the increases in the levels of H3K4me3 and SMYD3 in response to IL-4 stimulation were significantly attenuated in the presence of BCI-121 (50 µM) (Fig. 2c, Fig. S2).

### H3K4me3^high^ nasal epithelial cells induce the differentiation of cocultured naïve CD4^+^ T cells into Th2 cells

**Fig. 3:**
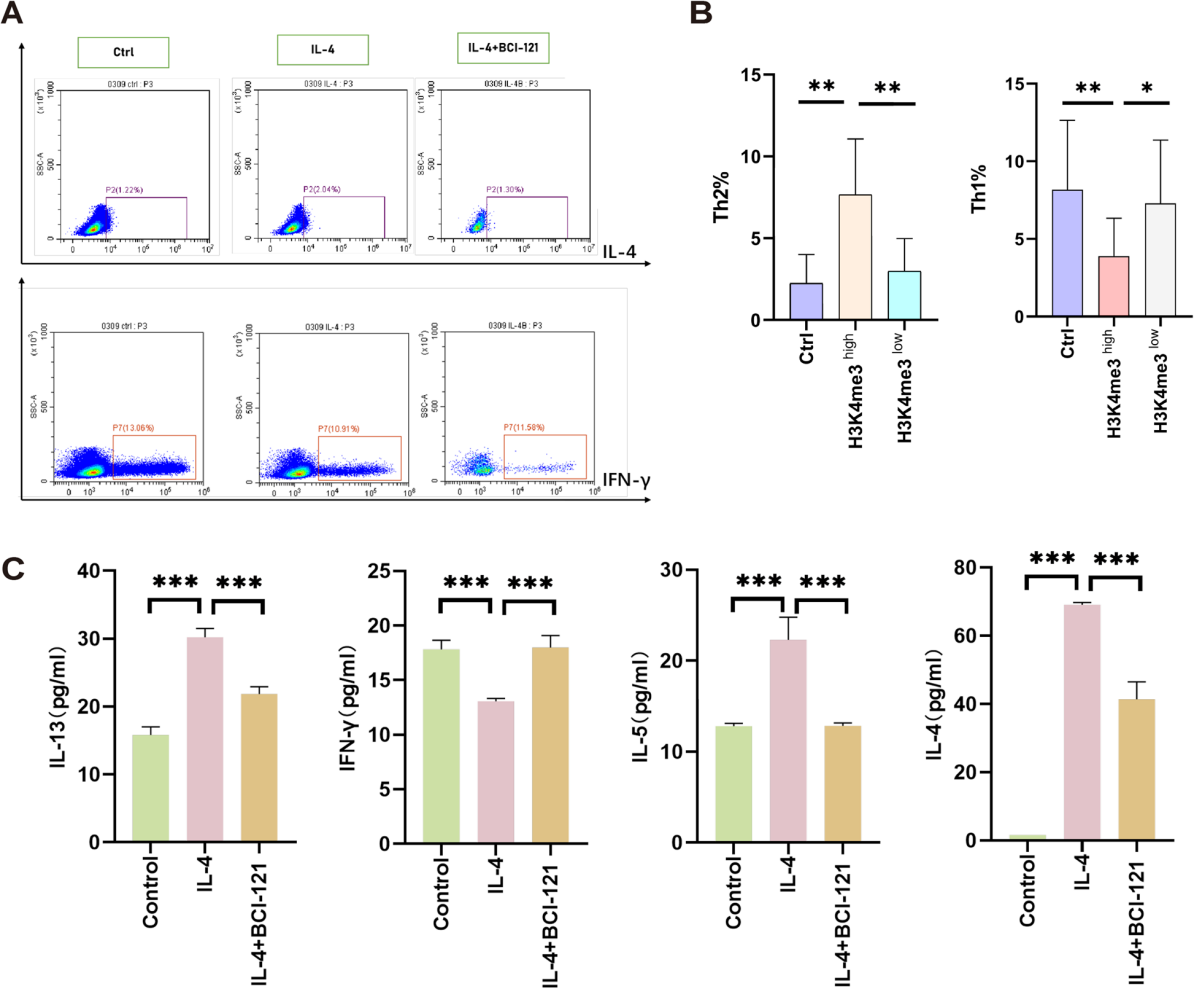
The SMYD3-mediated H3K4me3 modification level in primary human nasal epithelial cells influences the differentiation of cocultured naïve CD4 + T cells. **A**-**B** FACS results showing the percentage of Th2 cells in cocultures of primary human nasal epithelial cells pretreated with IL-4 in the presence or absence of BCI-121 for 72 h. The quantitative data are shown in (**B**) (*n* = 6 donors). **C** ELISA of IL-4, IL-5, IL-13 and IFN-γ secretion by cocultured naïve CD4 + T cells in the control group, IL-4 group and IL-4 stimulation + BCI-121 treatment group. Statistical significance (**B**-**C**), one-way ANOVA;***p < 0.001;**p < 0.01; ∗ *p* < 0.05

Previous studies [24] demonstrated that the level of histone modification in the airway epithelium can impact the local immune response and microenvironment. In the context of Th2 CRSwNP, the local Th2 polarization observed in the nasal mucosa may result from histone modification alterations in the nasal epithelium in the Th2-dominant microenvironment. Combined with this knowledge, we hypothesized that modifications in SMYD3-mediated H3K4me3 in the nasal epithelium play a role in promoting Th2 cell differentiation from naïve CD4 + T cells. To test this hypothesis, we conducted an experiment in which we cocultured primary nasal epithelial cells that were pretreated with IL-4 in the presence or absence of BCI-121 with naïve CD4 + T cells in a Transwell system. After 72 h, we determined the percentages of Th1 and Th2 cells using flow cytometry. The percentage of Th2 cells was increased in the IL-4-treated nasal epithelial cell group compared to the untreated group, while this increase was partially reversed when BCI-121 was added to the culture (Fig. 3a, b). We also measured the concentrations of Th1 and Th2 cytokines in the culture supernatants of the three groups by enzyme-linked immunosorbent assay (ELISA), and the results were consistent with the flow cytometry data (Fig. 3c).

These findings indicate that nasal epithelial cells with high SMYD3-mediated H3K4me3 levels may promote the differentiation of naïve CD4 + T cells into Th2 cells by secreting certain mediators. To identify the crucial mediator, we performed H3K4me3 chromatin immunoprecipitation followed by next-generation sequencing (ChIP-seq) to assess a range of N-terminal posttranscriptional modifications (marks), including H3K4me3, in nasal epithelial cells that were either untreated or stimulated with IL-4 and/or BCI-121.

### Th2 cell differentiation from naïve CD4 + T cells is enhanced by IGF2 produced by H3K4me3^high^ nasal epithelial cells pretreated with IL-4

**Fig. 4:**
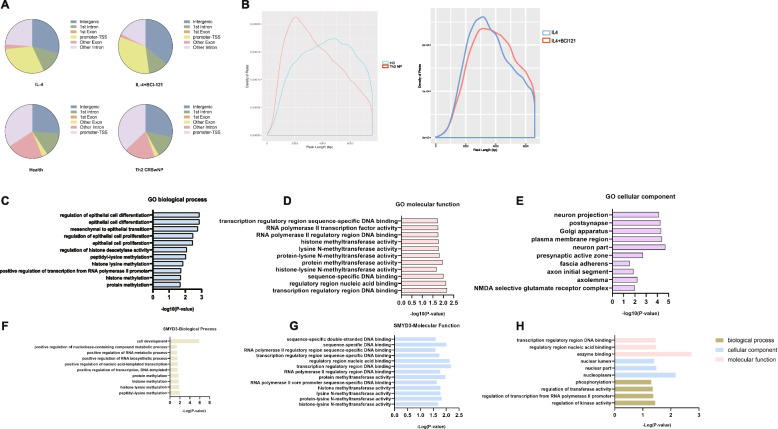
**A** Pie charts showing the distribution of H3K4me3 peaks across different genomic regions in human nasal epithelial cells and Th2 NP tissue. **B** Density of H3K4me3 peaks in the Th2 CRSwNP vs. HC and IL-4 vs. IL-4 + BCI-121 groups. **C**-**E** Top enriched GO terms for biological processes (BPs), molecular functions (MFs) and cellular components (CCs) associated with epithelial cells, HMT activity and transcription in the H3K4me3^high^ group. **F**-**G** Top enriched GO terms related to the specific gene SMYD3 in primary human nasal epithelial cells (IL-4 vs. control groups), including BP and MF terms. **H** GO term enrichment analysis of biological processes, cellular components, and molecular functions to assess the biological function of the SMYD3 gene in NPs from Th2 CRSwNP patients

The H3K4me3 ChIP-seq data revealed that the density and distribution of H3K4me3 peaks varied across genomic regions, including intergenic, 1st intron, 1st exon, other intron, other exon, and promoter-TSS regions. Notably, the distribution of H3K4me3 modifications exhibited a predominant focus on intergenic and promoter-TSS regions (Fig. 4a), and there were evident differences in the differential peaks in patients with Th2 CRSwNP compared with healthy controls, as well as in the IL-4 group compared with the IL-4 + BCI-121 group (Fig. 4b). Consistent with this pattern, from our functional analysis of the IL-4 H3K4me3^high^ group, Gene Ontology (GO) term enrichment analysis showed that IL-4 treatment induced upregulation of functions related to histone modification enzymes and epithelial changes (Fig. 4c-e). Regarding HMT activity, we analysed the molecular functions and biological processes related to the specific gene SMYD3 (Fig. 4f, g), and enrichment of GO:0000979 with SMYD3 was notable, along with robust H3K4me3 modification. There are two important GO terms regarding transcriptional regulation at the chromosome level, namely, GO:0003682 (chromatin binding) and GO:0000979 (RNA polymerase II core promoter sequence‐specific DNA binding), and the latter term shows enrichment of SMYD3. Moreover, most of the functions of SMYD3 are related to histone modifications and transcriptional activity, which might also explain from another perspective why SMYD3 functions as a specific H3K4me3 HMT impacting gene expression regulation in human nasal epithelial cells. However, the functions of SMYD3 did exhibit differences between NPs and nasal epithelial cells, partially due to the complex sources of SMYD3 (Fig. 4h). Detailed information is shown in Supplementary Tables 1, 2, 3, 4 and 5.

**Fig. 5:**
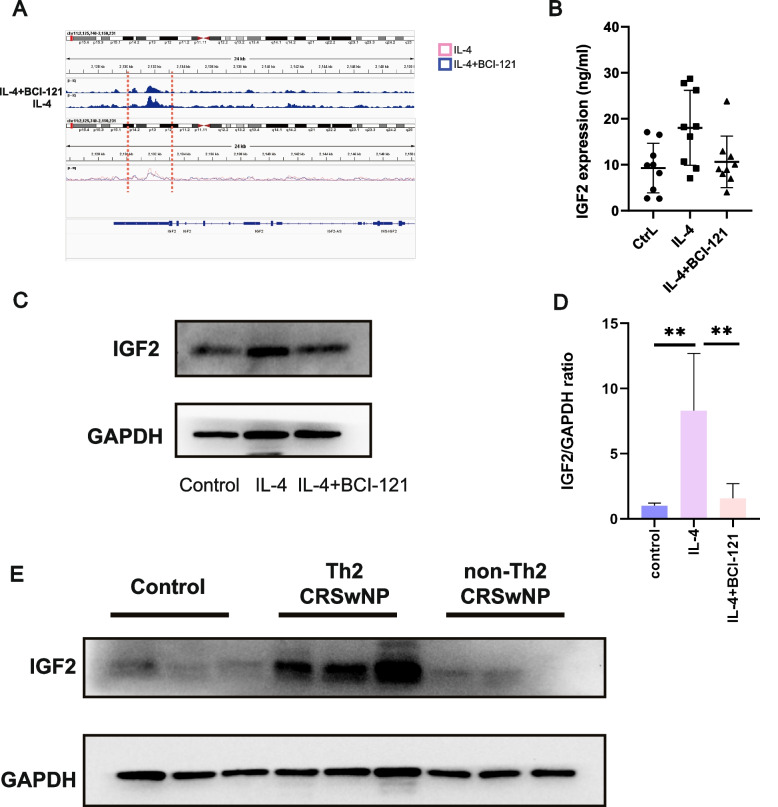
IL-4 stimulation increased the H3K4me3 level in IGF2 and increased IGF2 expression. **A** Integrated Genomics Viewer (IGV) visualization of the H3K4me3 enrichment status across the IGF2 gene, revealing that IL-4 promoted high H3K4me3 level-associated transcriptional activation of IGF2 in human nasal epithelial cells. The gene symbol is denoted at the bottom. **B**-**D** Primary human nasal epithelial cells with the SMYD3-mediated H3K4me3.^high^ status express high levels of IGF2, as determined by ELISA (**B**), immunoblotting (**C**) and quantitative RT‒PCR (**D**). **E** Immunoblot analysis of IGF2 protein expression in ITs from the control group and NPs from the Th2 CRSwNP and non-Th2 CRSwNP groups (*n* = 9). Statistical significance (**B** and **D**), one-way ANOVA;***p < 0.001;**p < 0.01; ∗ *p* < 0.05; ns, not significant (*p* > 0.05)

Moreover, Integrative Genomics Viewer (IGV) revealed higher enrichment of H3K4me3 on the IGF2 gene in the IL-4 group than in the IL-4 + BCI-121 group, and this enrichment was particularly prominent in the intergenic region (Fig. 5a). IGF2, a member of the growth hormone family involved in the immune pathogenesis of allergic diseases, is synthesized from an imprinted gene located in the p15.5 region on chromosome 11. It was found to be abundant in human nasal polyps by the Hartnell team [25]. Recently, the functions and roles of IGF2 in controlling T-cell differentiation, immune development and physiology have been acknowledged [26–30], suggesting that epithelial cell-derived IGF2 acts as a trigger, inducing the differentiation of naïve CD4 T cells into Th2 cells.

**Fig. 6:**
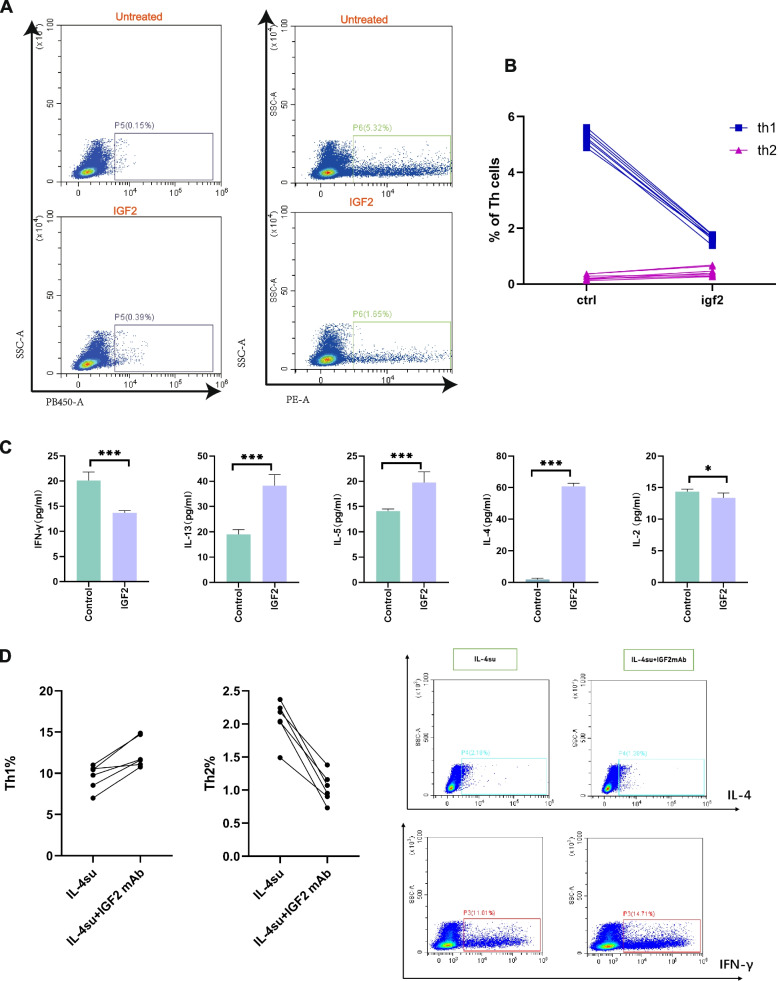
IL-4 induces an increase in SMYD3-mediated H3K4me3 modification of IGF2 expressed in primary human nasal epithelial cells to promote the differentiation of naïve CD4 + cells into Th2 cells. **A**-**B** IGF2 stimulation induces the differentiation of naïve CD4 + T cells into Th2 cells, as determined by FACS. **C** The concentrations of a range of secreted classical cytokines associated with Th2 and Th1 cells (IL-4, IL-13, IL-5, IFN-γ and IL-2) were measured with ELISA kits (mean ± s.d. *n* = 6). Statistical significance,Mann–Whitney U test;*** *p* < 10.^−3^;* *p* < 0.05; ns, not significant (*p* > 0.05). **D** IGF-2 derived from nasal epithelial cells induces the differentiation of naïve CD4 + T cells into Th2 cells, as determined by FACS (*n* = 6 donors)

Generally, H3K4me3 modification activates gene transcription, and enrichment of H3K4me3 is associated with high gene expression levels. We hypothesized that the IL-4-driven significant enrichment of H3K4me3 in IGF2 may promote IGF2 expression at both the translational and transcriptional levels. To investigate this possibility, we performed an IGF2 array analysis on the supernatant of cultured nasal epithelial cell. IL-4 stimulation increased the expression of IGF2 in epithelial cells. When BCI-121 was added, there was a moderate decrease in IGF2 expression compared to that in the group stimulated by IL-4 alone (Fig. 5b). Similarly, the expression of IGF2 in nasal epithelial cells was significantly increased at both the transcriptional and translational levels following IL-4 stimulation. Conversely, treatment with BCI-121 resulted in a noticeable decrease in IGF2 expression (Fig. 5 c, d). In addition, we assessed IGF2 protein expression in NPs and ITs and found that the expression of IGF2 protein was higher in NPs from the Th2 CRSwNP group and lower in NPs from the non-Th2 CRSwNP group than in NPs from the control group (Fig. 5e). Hence, we hypothesized that IGF2 may play a crucial role in inducing Th2 differentiation during Th2 CRSwNP development. To this end, we cocultured naïve CD4 + T cells with IGF2 for 72 h. Flow cytometric analysis revealed a higher proportion of Th2 cells in the IGF2 group than in the control group (Fig. 6a, b). Furthermore, naïve CD4 + T cells (na +) expressed high levels of IGF1R on their surface compared to other cells (na-) (Fig. S3), confirming that a receptor for the IGF2 ligand is expressed on naïve CD4^+^ T cell. Next, we measured the concentrations of IFN-γ, IL-4, IL-5, and IL-13 in the supernatants of naïve CD4^+^ T cells activated by IGF2 stimulation (Fig. 6c) and found that they were increased, consistent with the results of flow cytometric analysis. Furthermore, to test whether IGF-2 derived from nasal epithelial cells induces Th2 differentiation, we determined the proportion of Th2 cells after culture of naïve CD4^+^ T cells with supernatants from IL-4-treated nasal epithelial cells both in the presence and absence of xentuzumab (0.1 μmol/L, an IGF-2 neutralizing antibody). This analysis clearly showed that IGF-2 originating from nasal epithelial cells can induce Th2 differentiation, as depicted in Fig. 6d.

### The IL-4-driven SMYD3-mediated H3K4me3^high^ status of IGF2 in human nasal epithelial cells is mediated via the c-Myc-MAT2A axis

**Fig. 7:**
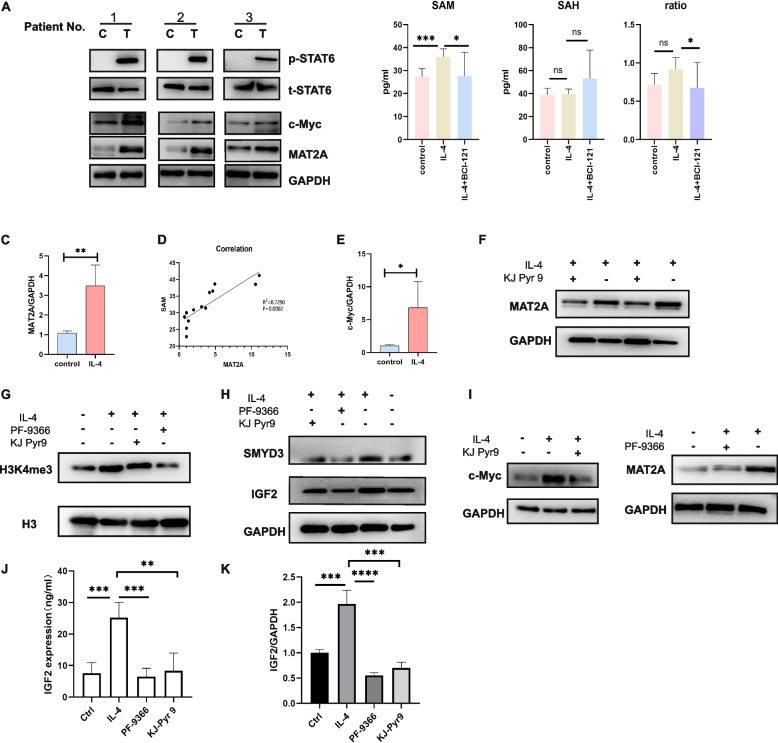
**A** Immunoblot analysis of the indicated proteins in the pathway of the SMYD3-mediated H3K4me3high status in primary human nasal epithelial cells induced by IL-4 (T) treatment compared with controls (**C**) (*n* = 3). **B** Effects of IL-4 and/or BCI-121 on the SAM level in nasal epithelial cells (n = 8 per group; error bars, s.d.) and SAH levels and SAM/SAH ratios in nasal epithelial cells (group *n* = 6 per group; error bars, s.d) (In the SAH experiment, two of the 8 samples were completely exhausted). **C** Quantitative RT‒PCR analysis of the MAT2A transcript level in human nasal epithelial cells stimulated with IL-4 for the indicated times (*n* = 4). **D** Positive correlation between the mRNA expression level of MAT2A and the level of SAM (*n* = 10), R.^2^ = 0.7770. **E** Quantitative RT‒PCR analysis of the c-Myc transcript level in human nasal epithelial cells stimulated with IL-4 for the indicated times (*n* = 4). **F** Immunoblot analysis of the effect of MAT2A expression on primary human nasal epithelial cells pretreated with IL-4 after exposure to the c-Myc inhibitor KJ Pyr 9 (10 μM). **G**-**H** Immunoblot analysis of H3K4me3, SMYD3 and IGF2 protein expression in primary human nasal epithelial cells pretreated with IL-4 after exposure to KJ Pyr 9 (10 μM) with or without PF-9366 (10 μM). **I** Immunoblot analysis of c-Myc and MAT2A protein expression in primary human nasal epithelial cells pretreated with IL-4 after exposure to KJ Pyr 9 (10 μM) with or without PF-9366 (10 μM). **J** ELISA of IGF2 as described in (H) (*n* = 4). **K** Quantitative RT‒PCR analysis of the IGF2 transcript level as described in (H) (*n* = 4). Statistical significance (**C** and **E**), unpaired Wilcoxon test; (**B** and **K**), one-way ANOVA;***p < 0.001;**p < 0.01; ∗ *p* < 0.05

Through in vitro studies, we investigated the mechanism underlying the IL-4-driven SMYD3-mediated H3K4me3^high^ status of IGF2 in primary human nasal epithelial cells. Our findings demonstrate that the activity of SMYD3 is a key factor in this process. First, the crosstalk between IL-4 and IGF2 mediated by signal transducer and activator of transcription 6 (STAT6) and phosphorylation of STAT6 has been demonstrated in immune cells [31, 32]. Because of the close relationship between IL-4 and p-STAT6, we performed Western blot analysis, and the results showed that IL-4 stimulation increased the level of p-STAT6 in nasal epithelial cells, supporting our hypothesis that the IL-4-induced robust production of IGF2 by nasal epithelial cells in the Th2-biased inflammatory milieu might rely on IL-4-mediated activation of p-STAT6 (Fig. 7a). As shown above, the upregulation of IGF2 expression was due to the H3K4me3^high^ status of IGF2, but the mediators linking IL-4/p-STAT6 to SMYD3-mediated H3K4me3 remain elusive. In our effort to identify the underlying pathway, SAM and methionine adenosyltransferase 2A (MAT2A) attracted our attention: the former serves as a principal methyl group donor for histone and DNA methylation during epigenetic regulation by regulating HMT activity [33–35], and the latter is an enzyme responsible for regulating SAM synthesis [36–38]. We observed that the concentration of SAM (Fig. 7b) and the expression of MAT2A were increased by IL-4 treatment in nasal epithelial cells (Fig. 7a, c). Moreover, there was a positive correlation between the SAM concentration and the MAT2A mRNA level (Fig. 7D), supporting the unique function of MAT2A related to SAM in IL-4-treated nasal epithelial cells. Consistent with published reports [39, 40], c-Myc, as a "bridge" linking MAT2A and IL-4/p-STAT6, might function as a mediator during this process, and its expression was found to be increased in the IL-4 group by Western blot analysis. Moreover, our Western blot and qRT‒PCR results also showed that IL-4 treatment induced upregulation of c-Myc (Fig. 7a, e). In support of the promotive effect of c-Myc on MAT2A expression, downregulation of MAT2A was observed after treatment with KJ Pyr 9 [41] an inhibitor of c-Myc, supporting the regulatory effect of c-Myc on MAT2A (Fig. 7f). Based on the above observations, we speculated that the c-Myc/MAT2A axis plays a crucial role in IL-4-driven SMYD3-mediated H3K4me3 modification by regulating SAM production. In confirmation of this hypothesis, the addition of either the c-Myc inhibitor KJ Pyr 9 (10 μM) or PF-9366 (10 μM), an inhibitor of MAT2A [42], to the IL-4-treated group resulted in decreased SMYD3 expression and decreased H3K4me3 modification, as shown by Western blotting (Fig. 7g, h), providing proof that MAT2A activity could affect SMYD3 activity [43]. Moreover, we quantified the effects of the two inhibitors, demonstrating by Western blotting that they affected the protein levels of their targets c-Myc and MAT2A (Fig. 7i). HMTs control histone modification levels, and SAM, a universal source of methyl groups for histone methylation, plays a vital role in mediating histone modification via HMT activity. Specifically, according to recent studies [34, 44–46], SAM might affect H3K4me3 modification more strongly than other types of histone modifications. In addition, SAM is linked to histone methylation via SMYD3, which transfers a methyl group from SAM to the histone substrate to result in H3K4me3, indicating a positive link between SAM and H3K4me3 modification. Next, we evaluated the levels of SAM and SAH and the SAM/SAH ratio under identical stimulatory conditions (Fig. 7b). IL-4 stimulation induced SAM secretion by epithelial cells, whereas exposure to BCI-121 resulted in a significant reduction in the SAM level. Both inhibitors decreased the level of SMYD3-mediated H3K4me3, which further decreased IGF2 expression at the translational and transcriptional levels (Fig. 7h, k). Consequently, SMYD3 inhibition led to a decrease in the H3K4me3 level by restricting SAM recruitment to histone sites, which in turn suppressed the H3K4me3 modification of IGF2, resulting in mitigation of Th2 polarization.

## Discussion

Epigenetic remodelling in the airway epithelium is closely associated with the inflammatory endotypes of chronic respiratory disorders, such as type 2 asthma [47] and CRSwNP [48]. In this study, we demonstrated that Th2 stimulation can result in increased levels of SMYD3-mediated H3K4me3 in the nasal epithelium, contributing to Th2 CRSwNP development. Moreover, we confirmed that the SMYD3-mediated H3K4me3^high^ status in the nasal epithelium is associated with the surrounding local Th2-biased inflammatory microenvironment. Furthermore, we revealed the mechanism by which overexpressed IGF2, secreted by nasal epithelial cells and dependent on the SMYD3-mediated H3K4me3^high^ status, is controlled by the c-Myc/MAT2A axis in the nasal epithelium. This mechanism promotes local differentiation of Th2 cells from naïve CD4 + T cells. The level of H3K4me3 in CRSwNP patients was previously evaluated by the authors [49]. However, one limitation of that study was the lack of further investigation into whether the H3K4me3 level differs across patients with various inflammatory endotypes of CRSwNP. The concept of the "CRSwNP endotype" has gained recognition worldwide, as it delineates distinct pathophysiological aetiologies and therapeutic approaches for CRSwNP based on the specific endotype in clinical settings. Our research findings highlight distinct patterns between Th2 CRSwNP and non-Th2 CRSwNP, revealing differential alterations in H3K4me3 modifications across the endotypes of CRSwNP. Understanding the correlations between H3K4me3 and endotypes of CRSwNP might provide novel insights for alleviating refractory Th2 CRSwNP and for identifying therapeutic approaches to manage refractory Th2 CRSwNP.

We acknowledge the presence of a complex and multifactorial network involving diverse types of histone modifications, not just H3K4me3, throughout the development of Th2 CRSwNP. It is essential to further investigate the involvement of other histone modifications in this process, as they could provide additional insights into the mechanisms underlying Th2 CRSwNP. For example, high levels of H3K9me3 and histone H3 lysine 18 (H3K18) acetylation in airway epithelium in the asthma group were found to be closely linked to mucosal inflammatory cell recruitment and remodelling processes [50–52], respectively. Additionally, histone H3 lysine 27 acetylation (H3K27ac) in the airway epithelium is closely associated with airway remodelling through the regulation of goblet cell differentiation [53]. These findings suggest that diverse types of histone modifications may exert unique effects during CRSwNP development. To gain a deeper understanding of the relationship between the histone modification status in the nasal epithelium and the pathogenesis of different CRSwNP endotypes, we can reveal new perspectives that contribute to defining the aetiology, diagnosing the condition, and providing effective therapeutic interventions.

Since the nasal epithelium is the first site of contact with inhaled environmental agents, it is well positioned for environmental factors to influence gene expression and ultimately disease susceptibility through epigenetic modifications. Here, we highlighted the impact of Th2-dominant microenvironment-induced alterations in the H3K4me3 status of IGF2 within the nasal epithelium on the local immune environment. However, importantly, our study has clear limitations regarding the identification of correlations between environmental factors and histone modifications in the nasal epithelium. The specific alterations in H3K4me3 within the epithelium triggered by external environmental stimuli, such as PM 2.5, bacterial byproducts, or metabolic factors, as well as the related mechanisms, remain unknown.

On the basis of recent studies on the functional roles of metabolism in epigenetic modification [54, 55], SAM metabolism is closely interconnected with histone enzymes by influencing methyl group metabolism. SMYD3 is a methyltransferase associated with SAM, linking SAM and H3K4me3 modification by transferring a methyl group from SAM to a histone substrate [56]. These findings suggest that cell metabolism is closely associated with the level of histone modifications in the nasal epithelium. Our data demonstrate that methyl group metabolism impacts the development of the IL-4-driven SMYD3-mediated H3K4me3high status in nasal epithelial cells and promotes Th2 differentiation. However, we acknowledge the possibility of an interactive association between SAM and SMYD3 activity, as there may be factors influencing both. Accurate quantification of the production of SAM for SMYD3 or histone methylation remains challenging. However, the changes in histone modifications observed in Th2 CRSwNP offer new insights into the therapeutic potential of targeting inflammation-induced histone modifications.

These data emphasize a role of IGF2 in the induction of Th2 polarization by the SMYD3-mediated H3K4me3^high^ status in the nasal epithelium and indicate that IGF2 might be a novel target for predicting or treating Th2 CRSwNP. On the basis of previous reports, IGF2 binding to IGF1R regulates CD4 T-cell differentiation [57–59], consistent with our data. Interestingly, IGF1R is also expressed in nasal epithelial cells [60]. However, whether IGF2 can directly influence the epithelium itself was not explicitly demonstrated in our study and requires further investigation. Due to the long-term surgical and medicinal treatments needed, patients with Th2 CRSwNP often have a large financial burden. Recently, there have been literature reports and clinical trials regarding various therapeutic strategies targeting IGF2/IGF1R, including neutralizing monoclonal antibodies or combinations of agents that block their activity, such as xentuzumab (BI836845) and teprotumumab [61–65]. The application of antibodies specific for IGF2 for Th2 CRSwNP may identify new targets for combination therapies to alleviate or prevent local Th2 inflammation.

## Materials and methods

### Reagents and antibodies

Detailed reagent and antibody information is provided in the Supplementary Reagents section.

### Histone lysine modifications and histone modification multiplex assay

**T**o extract total histones from NPs and ITs, the EpiQuik™ Total Histone Extraction Kit was used according to the manufacturer's protocol (Cat # OP-0006, EpiGentek). Th2 and non-Th2 CRSwNP diagnoses were made based on the levels of Th cytokines, including IL-4, IL-13, IL-17 and IFN-γ. In brief, frozen human tissues were weighed, cut into small pieces, homogenized with a homogenizer in 1X Pre-Lysis buffer, and centrifuged at 10,000 rpm for 1 min at 4 °C. Cells were centrifuged at 1000 rpm for 5 min at 4 °C, resuspended in 1X Pre-Lysis buffer for 10 min with gentle stirring and centrifuged at 10,000 rpm for 1 min at 4 °C. After the supernatant was removed, the pellets were resuspended in 100 μL of lysis buffer and incubated on ice for 30 min. The samples were centrifuged at 12,000 rpm for 5 min at 4 °C. Balance-dithiothreitol (DTT) buffer was added to each supernatant. Histone concentrations were quantified by measuring the OD values using a bovine serum albumin (BSA) standard. Multiple modifications on H3 were assessed using the EpiQuik Histone H3 Modification Multiplex Assay Kit (Cat # P-3100, EpiGentek) according to the manufacturer's instructions. In brief, histones were extracted as described above. Strip wells were coated with the corresponding capture antibodies, and the antibodies were detected with a detection antibody. Data were acquired via a microplate reader within 2 to 10 min at 450 nm with an optional reference wavelength of 655 nm. H3 modifications were quantified following the manufacturer's instructions.

### Western blot analysis

Protein concentrations in the lysates were determined using an enhanced BCA Protein Assay Kit. Equal amounts of protein from each sample were subjected to separation by SDS‒PAGE, electrophoretic transfer, immunoblotting and chemiluminescence detection as previously described [66]. Detailed information on the related antibodies is listed in the Supplementary Reagents section. Protein expression levels in the samples were normalized to those of GAPDH and H3.

### Primary human nasal epithelial cells and cell lines

Human nasal epithelial cells were brushed and collected as we previously described [67, 68]. In brief, primary nasal epithelial cells were collected from an Endobrush cytological sampling brush, passaged once in PneumaCult-Ex Medium (STEMCELL Technologies, Cologne, Germany) for differentiation, and then seeded on the polyester membranes of Transwell-Clear inserts (Corning, Lowell, Mass). When the cells were confluent, the medium in the apical chamber of the Transwell apparatus was removed, and the medium in the basal chamber was replaced with PneumaCult ALI Maintenance Medium (STEMCELL Technologies) for an additional 3 weeks for further analysis.

The human bronchial epithelial cell line BEAS-2B was obtained from the American Type Culture Collection (Manassas, VA, USA) and maintained in RPMI 1640 medium containing 10% foetal bovine serum (FBS; Thermo Fisher Scientific, Waltham, MA, USA), streptomycin (100 U/mL), and penicillin (100 U/mL) in a humidified incubator at 37 °C in an atmosphere of 5% CO2.

### Purification of naïve CD4 + T cells

PBMCs were isolated from healthy human volunteers (20–40 mL/person) as previously described [69]. Next, naïve CD4 T cells were purified from PBMCs by using a Naïve CD4 + T Cell Isolation Kit II following the manufacturer’s instructions [70, 71]. Naïve CD4 + T cells were cultured in RPMI 1640 medium at 37 °C in a humidified incubator with 5% CO2 to establish the coculture system.

### Stimulation of nasal epithelial cells with IL-4, establishment of the coculture model, and flow cytometry

**Fig. 8:**
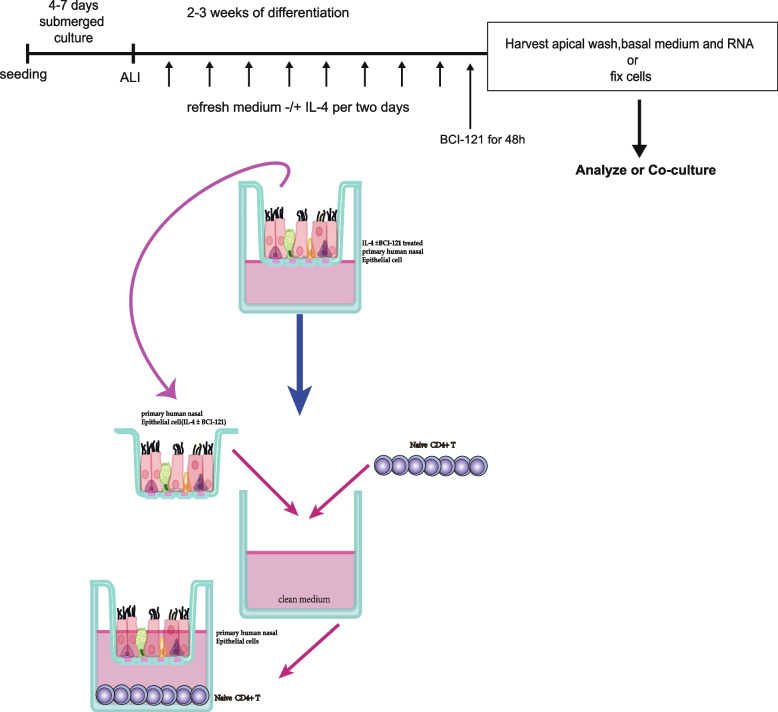
Schematic representation of the air–liquid interface (ALI) culture model of IL-4-human nasal epithelial cells, administration of BCI-121 and coculture with naïve CD4 + T cells. Primary human nasal epithelial cells were seeded onto Transwell membranes and allowed to grow to confluence for 4–7 days. Cells were exposed to air at the apical side (air–liquid interface; ALI) and stimulated for approximately 2–3 weeks at the basal side with medium (containing high concentrations of retinoic acid to induce mucociliary differentiation). To establish IL-4-treated human nasal epithelial cells, IL-4 was added to the basal medium upon the first exposure of the cells to air, and the medium with or without IL-4 was refreshed every 2 days for approximately 2–3 weeks (**A**). Then, when the nasal epithelial cells had differentiated, naïve CD4 + T cells were added to the basal medium with refreshment of IL-4-free medium and BCI-121-free medium (**B**)

Primary human nasal epithelial cells were cultured by using an air–liquid interface (ALI) method in a Transwell apparatus as previously mentioned [72]. In brief, the culture medium was refreshed every other day. Once the cells were completely confluent, the culture medium in the apical chamber was removed to allow further cell differentiation at the air–liquid interface (ALI) for 2–3 weeks, and the resulting epithelial cell cultures were used for in vitro studies. For the expression regulation experiments, human nasal epithelial cells from control subjects were cultured with the ALI method and treated with IL-4 and BCI-121. Nasal epithelial cells were incubated with IL-4 prior to coculture with naïve CD4 + T cells in RPMI 1640 medium (IL-4- and BCI-121-free). The detailed procedure is shown in Fig. 8a.

Naïve CD4 + T cells in complete medium were seeded immediately following isolation by density gradient centrifugation in the bottom compartments of Transwell chambers in 24-well plates (1.5 × 10^6^ cells/well). Cells were maintained in the coculture system for 72 h. A diagram of the coculture system is presented in Fig. 8b. Moreover, the supernatant in the bottom compartment of the Transwell plate was collected and assayed for secretion of the proteins of interest by ELISA, and cells were collected by flow cytometry to estimate the percentage of Th2 cells. Then, the cells were stained with fluorescently labelled primary antibodies and analysed by flow cytometry (Beckman CytoFlex S flow cytometer with CytExpert software) as reported previously [69]. Antibody information is provided in the Supplemental Reagents section.

### (ChIP assay and bioinformatics pipeline

All tissues were harvested for IP. The tissue preparation method and ChIP protocol closely adhered to the steps outlined in the SimpleChIP® Plus Sonication Chromatin IP System manual (Cell Signaling Technology, # 56383). For DNA quantification and qualification, genomic DNA degradation and contamination were monitored on 1% agarose gels. DNA purity was evaluated using a NanoPhotometer® spectrophotometer (IMPLEN, CA, USA). The DNA concentration was measured using a Qubit® DNA Assay Kit in a Qubit® 2.0 fluorometer (Life Technologies, CA, USA). For library preparation and quantification, 50 ng of DNA per sample was used as input material for ChIP sample preparation. Sequencing libraries were generated using the NEBNext® Ultra™ DNA Library Prep Kit for Illumina® (NEB, USA) following the manufacturer's recommendations, and indexing barcodes were added to attribute sequences to each sample. The library preparations were then sequenced on an Illumina platform, and 2 × 150 bp paired-end sequencing reads were generated.

ChIP sequencing library preparation and data analysis were conducted by LC-Bio (Hangzhou, Zhejiang 310018, China). Raw sequencing data were cleaned using FASTQC (v0.11.5) to filter out adapters and low-quality reads, and the unique mapped reads were collected by mapping the cleaned reads to a reference genome with no more than two mismatches by Bowtie 2. MACS2 (v2.1.1) was used to call peaks, resulting in robust and high-resolution ChIP-seq peak predictions. The peaks were annotated to the related genes using Homer (v4.10). deepTools (v2.4.1) was used to plot gene coverage of the reads near TSSs and transcription termination sites (TTSs). ChIPseeker (v1.5.1) was used to visualize the read distribution on chromosomes. Homer (v4.10) was used to search motifs and analyse transcription factors.

### qPCR

The detailed procedures for RNA isolation, complementary DNA (cDNA) synthesis, and qRT‒PCR analysis are reported in our earlier studies. In brief, total RNA was extracted with TRIzol reagent (Cat # 15596018, Invitrogen) according to the manufacturer’s instructions and was then used to synthesize first-strand cDNA using the 5X PrimeScript Kit (Cat # RR036A, Takara). Quantitative PCR was performed on the Applied Biosystems 7500 Standard Real-Time PCR System, and the relative expression of each gene was normalized to GAPDH expression. The primers used for gene amplification were as follows. MAT2A: (forward) 5'-ATGAACGGACAGCTCAACGG-3', (reverse) 5'-CCAGCAAGAAGGATCATTCCAG-3'; c-myc: (forward) 5'- AAGGTCAGAGTCTGGATCAC-3', (reverse) 5'- TAACTACCTTGGGGGCCTTT-3'; IGF2: (forward) 5'-'GTGGCATCGTTGAGGAGTG'-3', (reverse) 5'-GTGGCATCGTTGAGGAGTG'-3; GAPDH: (forward) 5'-AGATCATCAGCAATGCCTCCT-3', (reverse) 5'-TGAGTCCTTCCACGATACCAA-3'.

### ELISA

The IL-4, IL-13, IL-5, IL-2 and IFN-γ concentrations in the supernatants were measured using commercially available ELISA kits according to the manufacturer’s instructions. The IGF2 concentration was assessed with ELISA kits, and the SAM and SAH concentrations were quantified using commercially available ELISA kits.

### Single-nucleus RNA sequencing

Our team used frozen tissue as a source material to obtain purified nuclei for single-nucleus RNA sequencing. The Chromium Single Cell 3’ Kit V3 was used to construct libraries. The Illumina platform was used to perform sequencing. This analysis comprised two main processes (preprocessing pipeline, annotation and gene expression analysis), and the detailed procedures were described in previous publications [73, 74].

### Statistics

GraphPad Prism software for Windows (version 8.0; San Diego, CA, USA) was utilized for all statistical analyses in this study. Standard two-tailed, unpaired Student’s t test or one-way ANOVA was used for comparisons of parameters between two groups or among more than two groups, respectively. The data are expressed as the mean ± s.d. values. *P* < 0.05 and *P* < 0.01 were considered to indicate significant differences and highly significant differences, respectively. In vitro experiments were conducted at least two times with reproducibly consistent results (n ≥ 3 donors per group in each experiment).

### Samples and study approval

Adult patients with CRSwNP and control subjects were randomly recruited from the First Affiliated Hospital of Sun Yat-sen University. The study was endorsed by the Research Ethics Committee of Sun Yat-sen University. Tissues were obtained from the participants and divided into the control, Th2 and non-Th2 groups according to a panel (Fig. S1).

### Supplementary Information

**Additional file 1**.

**Additional file 2**.

**Additional file 3**.

**Additional file 4**.

**Additional file 5**.

**Additional file 6**.

## Data Availability

The datasets generated and/or analysed during the current study are available from the corresponding author upon reasonable request.
