# Supplementary material for: The SMYD3-dependent H3K4me3 status of IGF2 intensifies local Th2 differentiation in CRSwNP via positive feedback
Source: Cell Commun Signal. 2023 Nov 30;21:345. doi: 10.1186/s12964-023-01375-y (PMC10688075; doi:10.1186/s12964-023-01375-y)
Supplement: Supplementary file 6 — Additional file 5. [file 12964_2023_1375_MOESM5_ESM.docx]

| **REAGENT or RESOURCE** | **SOURCE** | **IDENTIFIER** |
| --- | --- | --- |
| **Antibodies** | | |
| Rabbit Anti-SMYD3 | Abcam | Cat#ab187149 |
| Anti-c-Myc | Abcam | Cat#ab32072 |
| Anti-STAT6 | Abcam | Cat#ab32520 |
| Anti-STAT6 (phospho Y641) | Abcam | Cat# ab12403 |
| Anti-Histone H3 (tri methyl K4) | Abcam | Cat# ab8580 |
| GAPDH | Cell Signaling Technology | Cat#5174S |
| Anti-Histone H3 | Abcam | Cat#ab195277 |
| Anti-IGF2 | Origene | Cat#TA327263S |
| Anti-MAT2A | Bioworld | Cat#BS71228 |
| Anti-rabbit IgG, HRP-linked Antibody | Cell Signaling Technology | Cat#7074S |
| **Chemicals, peptides, and recombinant proteins** | | |
| Naive CD4+ T Cell Isolation Kit II, human | Miltenyi Biotec | Cat#130-094-131 |
| TRIzol | Invitrogen | Cat# 15596018 |
| RPMI Medium 1640 | Gibco | C11875500BT |
| PrimeScript™ RT Master Mix (Perfect Real Time) | TAKARA | Cat# RR036A |
| TB Green® Premix Ex Taq™ | TAKARA | Cat# RR820A |
| PneumaCult-ALI Medium Kit 05001 | stemcell | 05001 |
| PneumaCult-Ex Medium 05008 | Stemcell | Cat# 05008 |
| KJ Pyr 9 | MCE | HY-19735 |
| PF-9366 | MCE | HY-107778 |
| Human IL-4 | PeproTech | Cat#200-04-20UG |
| Recombinant Human IGF-II | PeproTech | Cat#100-12-10ug |
| Protein Ladder | ThermoFisher | Cat#26616 |
| BCI-121 | MCE | Cat#HY-21972 |
| Xentuzumab | MCE | BI 836845 |
| EpiQuik Histone H3 Modification Multiplex Assay Kit (Colorimetric) | Epigentek | Cat#P-3100-96 |
| EpiQuik Total Histone Extraction Kit | Epigentek | Cat#OP-0006-100 |
| Brilliant Violet 421™ anti-human IL-4 | Biolegend | Cat#500826 |
| PE anti-human IFN-γ | Biolegend | Cat#506507 |
| PE anti-human CD221 (IGF-1R) | Biolegend | Cat#351806 |
| ELISA Kit for Insulin Like Growth Factor 2 (IGF2) | Cloud-clone | Cat#SEA051Hu |
| QuantiCyto® Human IL-4 ELISA kit | Neobioscience | Cat#EHC006.96 |
| QuantiCyto® Human IL-13 ELISA kit | Neobioscience | Cat#EHC137.96 |
| QuantiCyto® Human IFN-γ ELISA kit | Neobioscience | Cat#EHC102g.96 |
| QuantiCyto® Human IL-5 ELISA kit | Neobioscience | Cat#EHC148.96 |
| QuantiCyto® Human IL-2 ELISA kit | Neobioscience | Cat#EHC003.96 |
| Human SAM ELISA kit | Elabscience Biotechnoogy | Cat#E-EL-0043 |
| **Software and algorithms** | | |
| Prism | GraphPad | RRID: SCR_002798; http://www.graphpad.com/ |
| CytExpert software | Beckman Coulter | https://www.beckman.com/flow-cytometry/research-flow-cytometers/cytoflex/software |
| **Experimental models: Cell lines** | | |
| BEAS-2B(human bronchial epithelial cell line) | ATCC |  |
