# Supplementary material for: The SMYD3-dependent H3K4me3 status of IGF2 intensifies local Th2 differentiation in CRSwNP via positive feedback
Source: Cell Commun Signal. 2023 Nov 30;21:345. doi: 10.1186/s12964-023-01375-y (PMC10688075; doi:10.1186/s12964-023-01375-y)
Supplement: Supplementary file 7 — Additional file 6. [file 12964_2023_1375_MOESM6_ESM.docx]

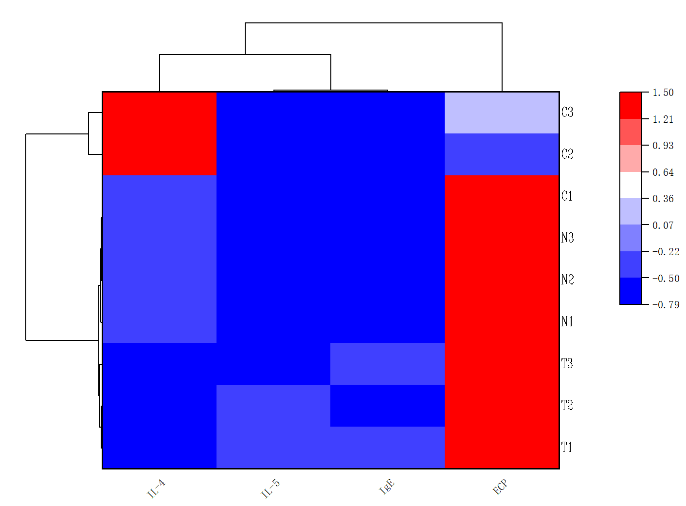


Figure.S1. The chart only presents information on the patients who participated in this study（n=9）.


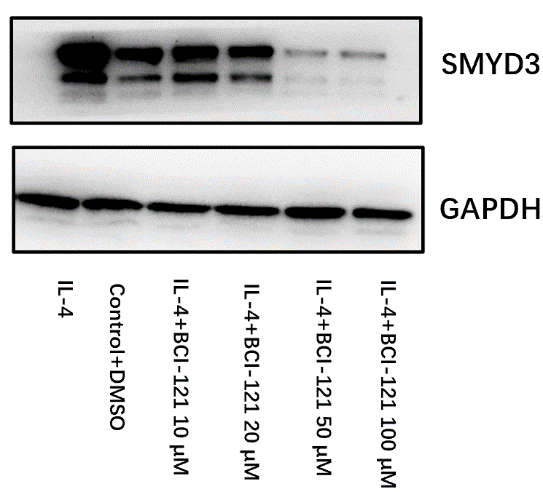


Figure.S2. Immunoblots of bulk SMYD3 in BEAS-2B cell line pretreated with IL-4 with or without the SMYD3 inhibitor BCI-121 containing diverse concentrations (0 to 100uM) within 48h. BCI-121 is a selective inhibitor for SMYD3.


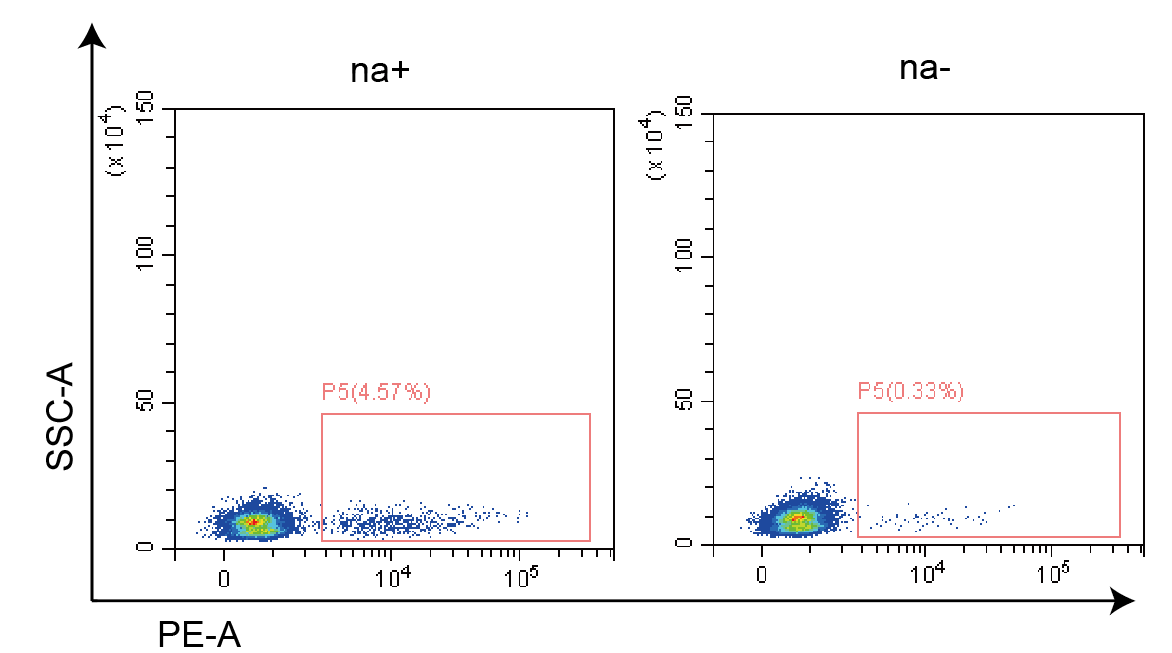

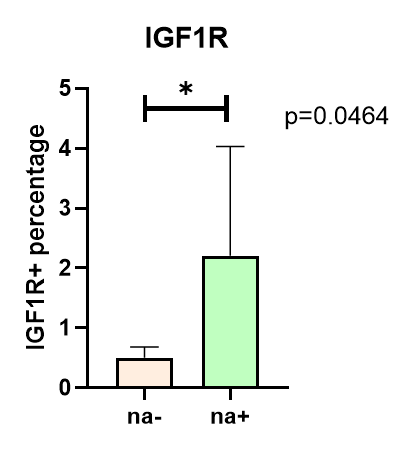


Figure.S3. FACS assay showing the IGF1R highly expresses in naïve CD4+ T cells. (B)Data in (A) is expressed as mean ± SD of (n=3) independent experiments. Statistical significance, unpaired Wilcoxon test，∗p < 0.05; ns, not significant (p > 0.05).


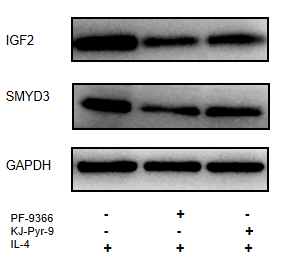


Figure.S4. Immunoblotting of H3K4me3, SMYD3 and IGF2 proteins in primary human nasal epithelial cells pretreated with IL-4 after exposure to KJ Pyr 9 (10 μM) and PF-9366 (10 μM) respectively,.
